# Cubebin, a Lignan Isolated from *Drimys andina*, Exhibits Potent and Selective Antiparasitic Activity against *Angiostrongylus cantonensis*

**DOI:** 10.1021/acsomega.5c05451

**Published:** 2025-07-11

**Authors:** Thainá R. Teixeira, Bernd Schmidt, Eric Sperlich, Bruna L. Lemes, Monique C. Amaro, Rebeca Pérez, Camilo Céspedes-Méndez, Cecilia Villegas, Viviana Burgos, Josué de Moraes, Cristian Paz

**Affiliations:** † Research Center on Neglected Diseases, 92928 Guarulhos University, Guarulhos, SP 07023-070, Brazil; ‡ Institut für Chemie, Universität Potsdam, Karl-Liebknecht-Str. 24-25, Potsdam D-14476, Germany; § Carrera de Química y Farmacia, Facultad de Ciencias de la Salud, Universidad Autónoma de Chile, Avenida Alemania 01090, Temuco 4780000, Chile; ∥ Departamento de Ciencias Biológicas y Químicas, Facultad de Recursos Naturales, Universidad Católica de Temuco, Rudecindo Ortega, Temuco 4780000, Chile; ⊥ Escuela de Tecnología Médica, Facultad de Salud, Universidad Santo Tomás, Temuco 4780000, Chile; # Research Center on Neglected Diseases, Scientific and Technological Institute, Brazil University, São Paulo, SP 08230-030, Brazil; ∇ Laboratory of Natural Products & Drug Discovery, Center CEBIM, Department of Basic Sciences, Faculty of Medicine, 28057 Universidad de La Frontera, Temuco 4780000, Chile

## Abstract

*Angiostrongylus cantonensis* is a zoonotic parasitic nematode of growing global health concern, largely due to the limited efficacy of current anthelmintics such as albendazole. In this study, cubebin—a dibenzylbutyrolactole lignan—was isolated for the first time from *Drimys andina* (Winteraceae), a Chilean endemic plant, and evaluated for its antiparasitic activity. Chromatographic purification of fresh leaves yielded cubebin as a 3:2 epimeric mixture, with its structure confirmed by 500 MHz NMR and single-crystal X-ray diffraction. *In vitro* assays demonstrated potent anthelmintic activity against both first-stage (L1) and infective third-stage (L3) larvae of *A. cantonensis*, with EC_50_ values of 4.7 and 15.3 μM, respectively, making it approximately three times more potent than albendazole against L1 and comparably effective against L3. Cubebin exhibited no cytotoxicity toward monkey (Vero) or human (HaCaT) cell lines and no toxicity in *Caenorhabditis elegans*, indicating a favorable safety profile. *In silico* ADME analysis further revealed favorable pharmacokinetic and drug-likeness properties. These results highlight cubebin as a promising lead compound for the development of novel anthelmintic therapies targeting *A. cantonensis* and potentially other parasitic nematodes.

## Graphical abstract

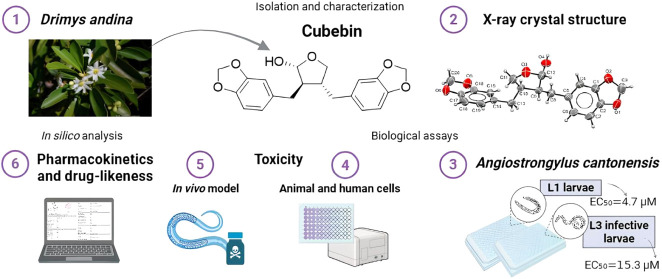

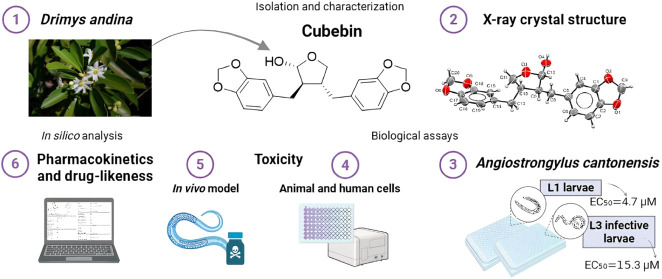

## Introduction

Parasitic nematode infections represent a major global health burden, affecting millions of humans and livestock worldwide.^1,2^ Among them, *Angiostrongylus cantonensis* (rat lungworm) stands out as an emerging zoonotic agent of clinical relevance.^3^ Human infection occurs accidentally through ingestion of raw or undercooked snails, slugs, or paratenic hosts (e.g., shrimp, frogs, lizards, crabs), or vegetables contaminated with mucus from infected mollusks.^4^ In humans, the parasite fails to complete its life cycle and instead migrates to the central nervous system, where it dies and triggers a pronounced inflammatory response, leading to eosinophilic meningitis (neuroangiostrongyliasis).^4,5^

Current treatment options for nematode infections, such as albendazole, show reduced efficacy against tissue-migrating larval stages.^2,3^ These limitations highlight the urgent need for safer and more effective anthelmintic alternatives. In addition to its clinical importance, *A. cantonensis* serves as a valuable experimental model due to its physiological similarity to other parasitic nematodes and its suitability for standardized *in vitro* screening assays.^6–9^

Natural products, particularly lignans, have shown promise as antiparasitic agents due to their distinct mechanisms of action and reduced risk of cross-resistance.^10,11^ *Drimys andina* (Winteraceae), a Chilean endemic species native to the Andes Mountains,^12^ has previously been reported to contain flavonoids and drimane sesquiterpenoids.^13,14^ However, to the best of our knowledge, cubebin has not been previously isolated from this species.

Cubebin is a known dibenzylbutyrolactone lignan previously identified in members of the Aristolochiaceae,^15–17^ Piperaceae,^18^ and several other botanical families.^19–23^ It has demonstrated a wide range of biological activities, including antiprotozoal,^17,24^ neuroprotective,^25^ anti-inflammatory,^26^ and vasomodulatory effects,^27^ making it an attractive candidate for further pharmacological evaluation.

In this study, we report the first isolation of cubebin from *D. andina* and evaluate its *in vitro* anthelmintic activity against first-stage (L1) and infective third-stage (L3) larvae of *A. cantonensis*. We compare its efficacy to that of albendazole and assess its safety in mammalian cells and *Caenorhabditis elegans*. *In silico* ADME analysis was also conducted to evaluate its pharmacokinetic and drug-likeness properties. Collectively, our findings support cubebin as a promising lead compound for the treatment of neuroangiostrongyliasis and potentially other helminthic infections.

## Results

### Isolation and Structural Analysis of Cubebin

A total of 7151 g of leaves from *Drimys andina* (Figure 1) were dried at 50 °C producing 2860 g of dried material (60% humidity). After purification by CC 168 mg cubebin (00,023% yield based on humid material) was recovered. Cubebin was obtained as colorless crystals. High-resolution mass spectrometry (HREIMS) confirmed the molecular formula C_20_H_20_O_6_ ([M^+^] calcd: 356.1254; found: 356.1252). Full ^1^H and ^13^C NMR assignments (Table S1) were consistent with previously published data for cubebin isolated from *Piper cernuum* and *Aristolochia* species.^15,28^

**Figure 1:**
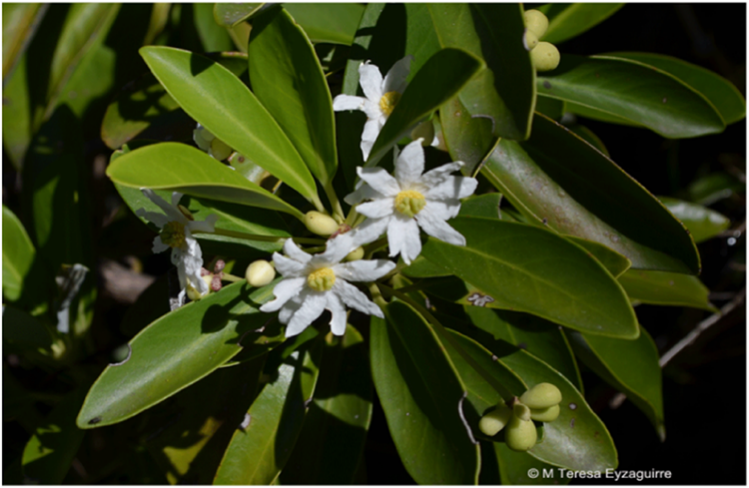
*D. andina* (Reiche) R.A Rodr. and Quez (Winteraceae), photographed in its native habitat.

### Molecular Structure of Cubebin

Cubebin (Figure 2) is a dibenzylbutyrolactole lignan. Its molecular structure was confirmed by NMR-spectroscopic analysis at 500 MHz; all data match those previously reported in the literature very well (Table S1 and Figures S1–S7). In CDCl_3_, cubebin is present as a 3:2 mixture of epimers, as determined by integration of the signals observed for the protons at position 9′ (Figure 2).

**Figure 2:**
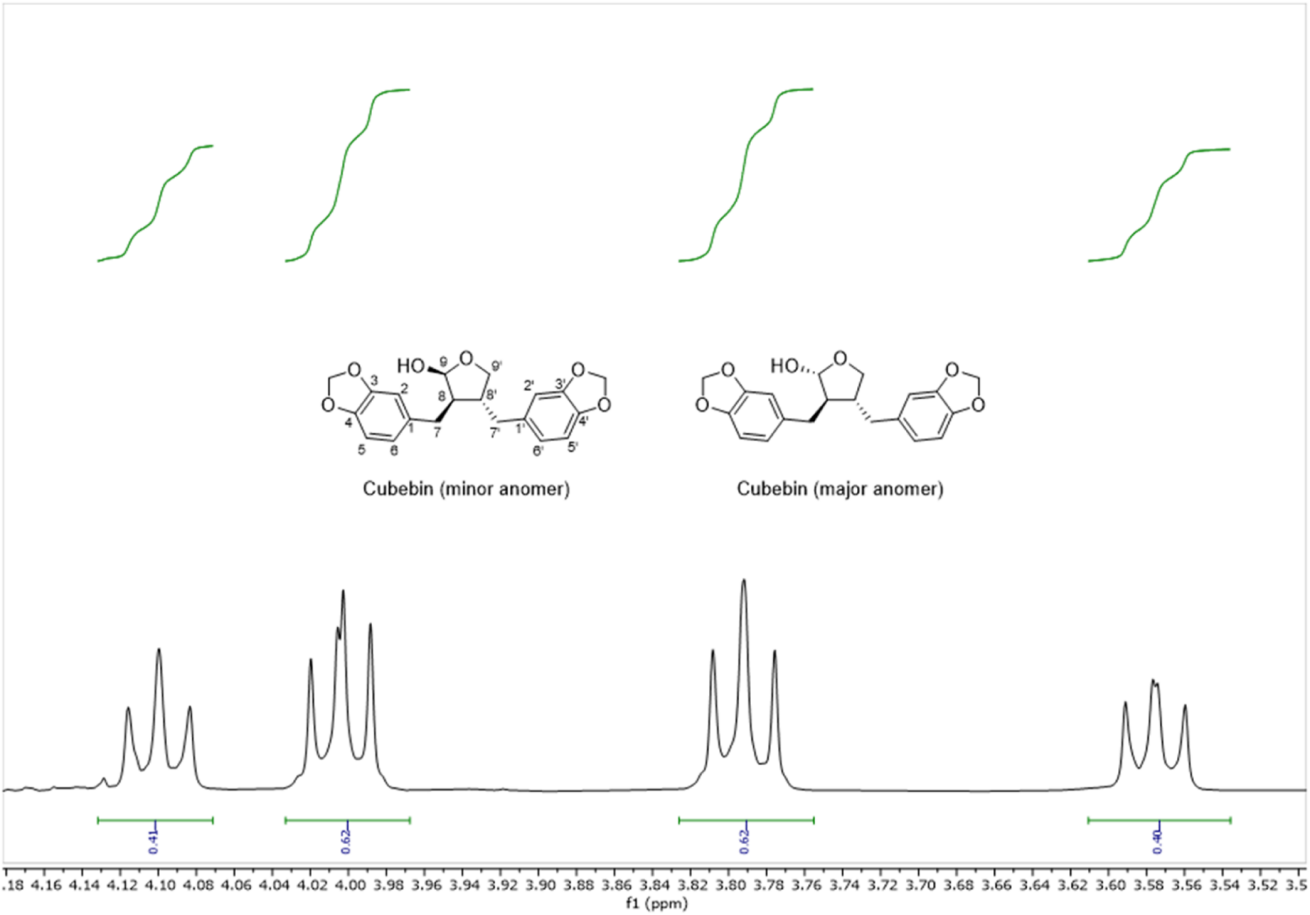
Signals for protons 9′ for minor and major anomer of cubebin. The anomeric ratio was determined by integration of the corresponding signals.

In addition, the molecular structure of cubebin was determined by single crystal X-ray analysis. A single crystal X-ray structure determination has earlier been reported by Macedo et al.,^28^ who also determined the absolute configuration by crystallography. In the solid state, only one epimer is observed, exhibiting a cis-configuration between the C9 hydroxyl group and the benzyl substituent at C8. This corresponds to the minor epimer in solution (Figure 3). Complete crystallographic data are provided in Table S2 and Figures S8–S13.

**Figure 3:**
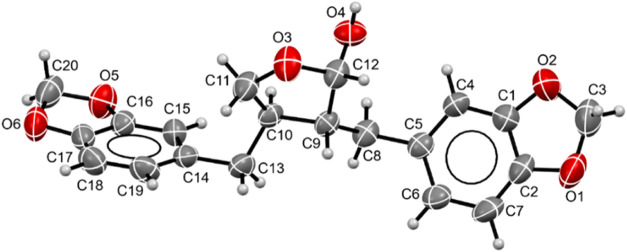
X-ray crystal structure of cubebin. Displacement ellipsoids shown at 50% probability level.

### Biological Studies

Cubebin’s anthelmintic activity was evaluated against first-stage (L1) and infective third-stage (L3) larvae of *A. cantonensis*, alongside cytotoxicity assessments in mammalian cell lines and toxicity profiling in *C. elegans*. Results are summarized in Table 1 and Figures 4 and 5.

**Figure 4:**
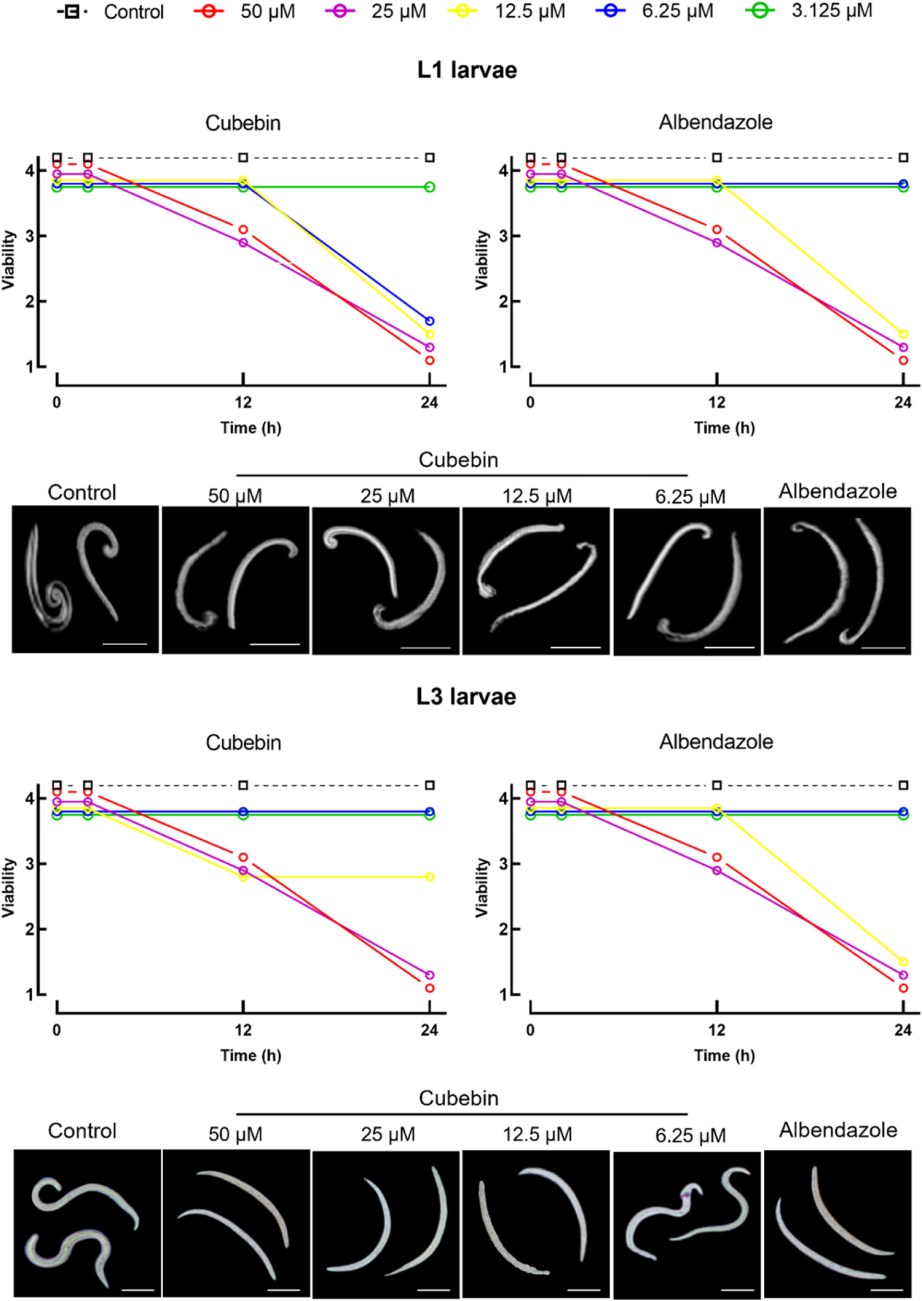
Time-dependent larvicidal activity and morphological effects of cubebin and albendazole on *A. cantonensis* larvae. Larval viability scores (1 = immotile to 4 = highly active) for first-stage (L1) and infective third-stage (L3) larvae treated with cubebin or albendazole at 2, 12, and 24 h. Representative micrographs of L1 and L3 larvae after 24 h of exposure to cubebin at the indicated concentrations, albendazole (12.5 μM), or negative control. Note the preserved caudal morphology in larvae treated with cubebin and albendazole compared to the pronounced caudal contortion observed in the control group. Images were acquired using a Motic AE2000 inverted microscope. Scale bars: 270 μm for L1 larvae; 400 μm for L3 larvae.

**Figure 5:**
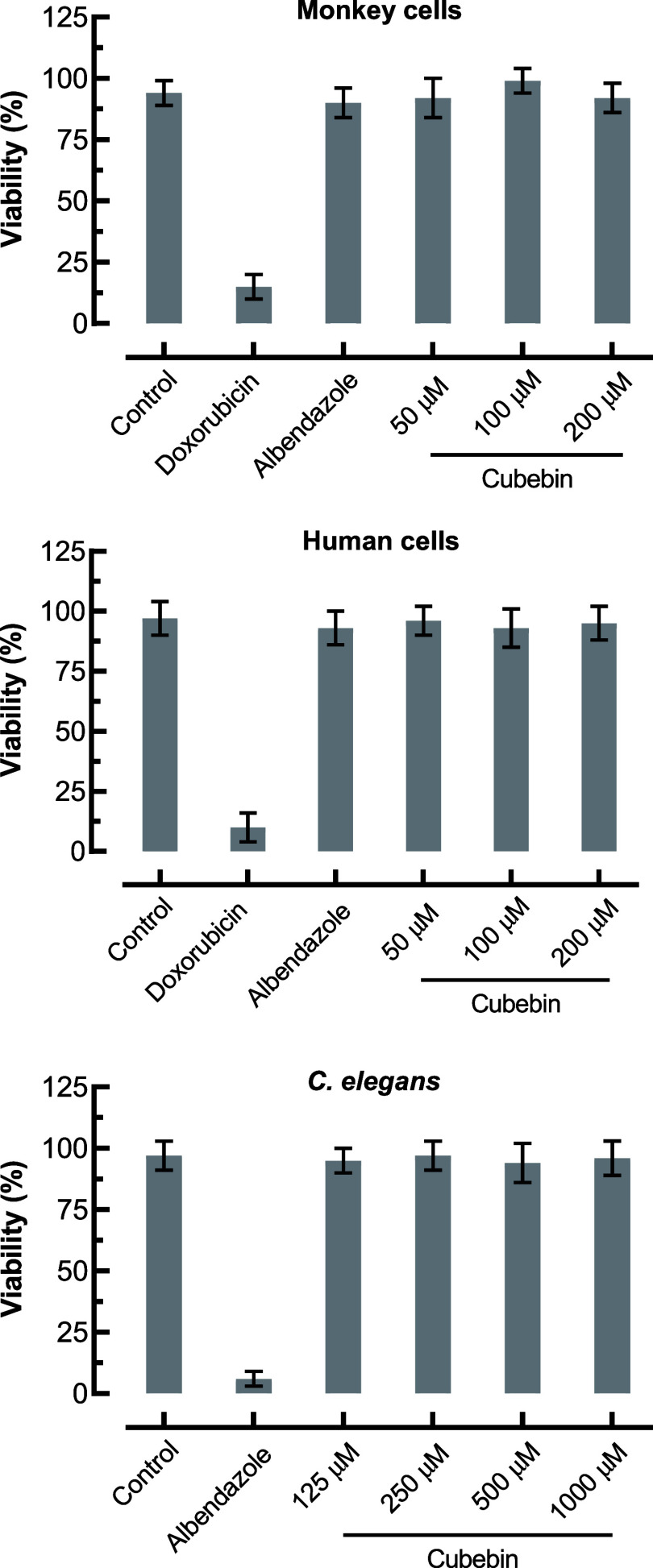
Toxicity assessment of cubebin in mammalian cells and *C. elegans*. *In vitro* cytotoxicity of cubebin (1–200 μM) and albendazole (200 μM) was assessed in Vero (monkey) and HaCaT (human) cell lines after 48 h of exposure. Doxorubicin (10 μM) served as the positive control for cytotoxicity, and vehicle-treated cells (0.5% DMSO) were used as the negative control. *In vivo* toxicity was evaluated in *C. elegans* after 72 h of exposure to cubebin (1–1000 μM), with albendazole (15 μM) as the nematicidal positive control and vehicle-treated worms as the negative control. Data represent mean ± SD (*n* = 3).

**Table 1:** Anthelmintic Activity and Toxicity Profile of Cubebin and Albendazole^a^ ^,^ ^b^ ^,^ ^c^ ^,^ ^d^ ^,^ ^f^

	*A. cantonensis* EC_50_ (μM)		mammalian cells CC_50_ (μM)		selectivity index		*C. elegans* LD_50_ (μM)
compound	L1	L3	monkey	human	L1	L3	
cubebin	4.7 ± 0.8^e^	15.2 ± 0.9	>200	>200	>42.5	>13.1	>1000
albendazole	12.8 ± 1.3	12.3 ± 1.9	>200	>200	>15.6	>16.2	13.6 ± 1.1

a EC_50_: Effective concentration 50%.

b CC_50_: Cytotoxic concentration 50%.

c LD_50_: lethal concentration 50%.

d Selectivity index (SI) was calculated as SI = CC_50_ (human HaCaT cells)/EC_50_.

e *P* < 0.01 compared to albendazole.

f Data are expressed as mean ± standard deviation (SD) from three independent experiments.

#### Anthelmintic Activity of Cubebin against L1 and L3 Larvae of *A. cantonensis*

Cubebin exhibited significant larvicidal activity against first-stage (L1) larvae of *A. cantonensis*, with an EC_50_ of 4.7 ±  0.8 μM—approximately 2.7 times more potent than albendazole (EC_50_ = 12.8 ±  1.3 μM). Against infective third-stage (L3) larvae, cubebin showed an EC_50_ of 15.2  ± 0.9 μM, which was comparable to that of albendazole (EC_50_ = 12.3  ±  1.9 μM). The overlapping confidence intervals suggest no statistically significant difference in efficacy at this stage (Table 1). These results indicate that cubebin is particularly effective against early larval stages while maintaining efficacy similar to albendazole at the infective stage.

#### Time-Dependent Larvicidal Effects and Morphological Analysis

Time-course analysis revealed rapid larvicidal activity for both cubebin and albendazole. A marked decline in larval viability was observed between 12 and 24 h postexposure, as assessed by motility scoring (Figure 4). By 24 h, cubebin induced complete immobility (score = 1) in both L1 and L3 larvae, mirroring the effects of albendazole.

Light microscopy revealed distinct morphological responses following treatment. Both L1 and infective L3 larvae exposed to cubebin or albendazole retained normal caudal morphology, with no evidence of caudal contortion. In contrast, pronounced caudal contortion was consistently observed in untreated controls and in larvae exposed to sublethal concentrations, suggesting a dose-dependent effect on larval morphology (Figure 4).

#### Toxicity and Selectivity

Cubebin exhibited no cytotoxicity toward monkey (Vero) or human (HaCaT) cell lines, with CC_50_ values exceeding 200 μM in both assays (Table 1 and Figure 5). These results yielded high selectivity indices (SI = CC_50_/EC_50_), with values of >42.5 for L1 larvae and >13.1 for L3 larvae, indicating a favorable therapeutic window.

Albendazole also showed no cytotoxicity at concentrations up to 200 μM. However, its selectivity index for L1 larvae was lower (>15.6) due to its reduced efficacy, while its SI for L3 larvae (>16.2) was comparable to cubebin, reflecting similar potency at this stage.

Importantly, cubebin displayed no observable toxicity in *C. elegans* at concentrations up to 1000 μM, further supporting its safety profile. In contrast, albendazole exhibited potent toxicity in *C. elegans*, consistent with its broad-spectrum nematicidal activity.

#### *In Silico* Drug-Likeness and ADME Profiling

Cubebin demonstrated favorable pharmacokinetic and drug-like properties critical for therapeutic development (Table 2). The compound adheres to all major drug-likeness rules, including Lipinski’s Rule of Five (molecular weight = 356.12 Da, log *P* = 3.18), with a polar surface area (TPSA = 66.38 Å^2^) and aqueous solubility (log *S* = −4.26) supporting membrane permeability and oral bioavailability.

**Table 2:** *In Silico* ADME, Physicochemical, and Drug-Likeness Properties of Cubebin^a^

parameters	cubebin
molecular weight (Da)	356.12
TPSA (Å)	66.38
log *P*_o/w_	3.18
log *S*	–4.26
gastrointestinal absorption	high
blood-brain barrier permeation	yes
P-gp substrate	no
Lipinski (Pfizer)	yes
Ghose (Amgen)	yes
Veber (GSK)	yes
Egan (Pharmacia)	yes
Muegge (Bayer)	yes
bioavaliability Score	0.55
PAINS	0 alert
brenk	0 alert

a Summary of absorption, distribution, metabolism, and excretion (ADME) parameters, along with physicochemical and drug-likeness evaluations of cubebin, as predicted by the SwissADME platform. TPSA: topological polar surface area; log Po/w: octanol–water partition coefficient; log S: aqueous solubility; P-gp: P-glycoprotein. Bioavailability and drug-likeness were assessed using filters established by Lipinski (Pfizer), Ghose (Amgen), Veber (GSK), Egan (Pharmacia), and Muegge (Bayer). PAINS: pan-assay interference compounds; Brenk: structural toxicity alerts.

Bioavailability radar analysis (Figure S14) further confirmed cubebin’s optimal drug-like properties, with all six parameters—lipophilicity, size, polarity, solubility, flexibility, and saturation—residing within the ideal range for oral bioavailability. Computational predictions indicated high gastrointestinal absorption (95% probability) and blood-brain barrier (BBB) penetration (Table 2).

Importantly, cubebin was not predicted to be a P-glycoprotein (P-gp) substrate, suggesting a low risk of efflux-mediated resistance. The compound also passed PAINS and Brenk filters, indicating the absence of structural alerts for toxicity or assay interference (Table 2).

## Discussion

This study reports, for the first time, the isolation of cubebin from *D. andina*, a Chilean endemic species in the Winteraceae family. While cubebin has been previously identified in members of the *Piperaceae* and *Aristolochiaceae* families, its presence in *Drimys* species had not been documented. This novel finding expands the known phytochemical repertoire of *D. andina* and reinforces its potential as a source of bioactive natural products.

These results establish *D. andina* as a new natural source of cubebin, a dibenzylbutyrolactone lignan that exhibited potent anthelmintic activity against *A. cantonensis* larvae. This aligns with a growing body of evidence highlighting the therapeutic potential of lignans and neolignans across plant taxa. For example, *Drimys brasiliensis* produces secondary metabolites with activity against *Schistosoma mansoni*,^29^ while cubebin-rich *Piper cubeba* fruits exhibit larvicidal activity against *Hemonchus contortus*.^30^ Similarly, neolignans such as licarin A from *Lauraceae* species and those from *Saururus cernuus* (*Saururaceae*) demonstrate potent schistosomicidal effects with low cytotoxicity.^10,11^ These findings support the broader utility of lignans as promising antiparasitic scaffolds with favorable safety profiles.^31,32^

Cubebin exhibited stage-specific anthelmintic activity, demonstrating 2.7-fold greater potency than albendazole against L1 larvae of *A. cantonensis*, while maintaining comparable efficacy against infective L3 larvae. These findings suggest that cubebin may act through a mechanism distinct from that of benzimidazoles, which exert their effects by disrupting microtubule polymerization.^2^ Notably, morphological analysis showed no signs of caudal contortion in larvae treated with either cubebin or albendazole, indicating that cubebin’s mode of action likely involves a different pathway than the cytoskeletal damage typically associated with benzimidazoles.

Toxicity profiling confirmed cubebin’s favorable safety profile in mammalian cells (Vero and HaCaT), with no cytotoxicity observed at concentrations up to 200 μM. The resulting selectivity indices were high—greater than 42.5 for L1 larvae and 13.1 for L3 larvae of *A. cantonensis*—supporting a wide therapeutic window. These findings are consistent with reports on other lignans, such as licarin A and neolignans from *S. cernuus*, which also exhibit potent antiparasitic activity with low mammalian cytotoxicity.^10,11^ Cubebin was likewise nontoxic to *C. elegans*, a widely used *in vivo* model for early-stage toxicity screening.^33^ Interestingly, lignans from *Arctium lappa* have been shown to extend *C. elegans* lifespan *via* antioxidant mechanisms,^34^ suggesting that cubebin’s lack of toxicity may be associated with cytoprotective properties rather than pharmacological inertness.

*In silico* ADME and drug-likeness analysis further support cubebin’s potential as a drug candidate. The compound complies with key criteria from Lipinski, Veber, and Ghose rules, demonstrating favorable molecular weight, lipophilicity, polar surface area, and solubility. It exhibited high predicted gastrointestinal absorption (95%) and blood-brain barrier permeability—key properties for treating neuroangiostrongyliasis. Cubebin was not identified as a P-glycoprotein substrate, reducing the risk of efflux-mediated drug resistance, and it passed all PAINS and Brenk filters, indicating low risk of off-target effects. These pharmacokinetic and safety characteristics are consistent with other lignans like licarin A, which also combine favorable ADME profiles with potent antiparasitic activity.^10,35^ Taken together, these computational findings align with the experimental safety data, supporting cubebin as a viable lead compound for further development.

Interestingly, the absence of drimane sesquiterpenoids—well-documented in *Drimys winteri*—in *D. andina* leaves suggests ecological or genetic divergence in secondary metabolite production within the genus. Whereas *D. winteri* relies on sesquiterpenes for its bioactivity,^14,36^ *D. andina* appears to favor lignans such as cubebin. This divergence underscores the untapped pharmacological potential of *D. andina*, aligning with increasing interest in neolignans as leads for drug discovery, particularly due to their structural diversity and low risk of toxicity.^37^

In conclusion, cubebin emerges as a promising anthelmintic lead, exhibiting high selectivity, stage-specific efficacy, and favorable drug-likeness. Its identification in *D. andina* enriches the phytochemical landscape of the Winteraceae family and reinforces the importance of lignans in antiparasitic drug discovery. Future work should focus on evaluating cubebin’s *in vivo* efficacy, elucidating its mechanism of action, and exploring synergistic potential with existing treatments to address emerging drug resistance in helminths.

## Conclusions

This study presents cubebin as a promising anthelmintic agent, isolated for the first time from *D. andina*, a Chilean endemic species. Cubebin demonstrated potent and selective activity against *A. cantonensis* larvae, surpassing the efficacy of albendazole at early developmental stages while maintaining a favorable safety profile in mammalian cells and *C. elegans*. *In silico* pharmacokinetic and drug-likeness analyses further support cubebin’s potential for oral bioavailability and blood-brain barrier penetration—key features for treating neuroangiostrongyliasis. Together, these findings support cubebin’s advancement as a lead compound for anthelmintic drug development and highlight *D. andina* as a valuable source of bioactive lignans for parasitic disease control.

## Materials and Methods

### General Information

Column chromatography was performed using Merck silica gel 60 and Sephadex LH-20 (25–100 μm; Aldrich, Santiago, Chile). The progress of purification was followed by using analytical thin-layer chromatography (TLC) from Merck Silica Gel 60F254 sheets (Darmstadt, Germany), together with Low-Field NMR (LF-NMR, Bruker 80 Benchtop, Rheinstetten, Germany). TLC was eluted with a mixture of solvents as *n*-hexane (hex) and ethyl acetate (EtOAc) evaluated by UV light (254 nm) and then stained with KMnO_4_. Solvents and fractions were concentrated in a rotavap Büchi R100 at 45 °C. Solvents used in this study were distilled prior to use and dried over appropriate drying agents.

### Extraction and Purification of Cubebin

A total of 7151 g of fresh *D. andina* leaves of various sizes were collected in Huincacara, Villarrica, located in the Araucanía Region of Chile (coordinates: 39°28′54.9″S, 71°45′24.3″W; altitude: 1438 m above sea level), during the summer month of January 2024. A voucher specimen was deposited at the herbarium of the Universidad de La Frontera (DA-01–2024). The plant material was washed, oven-dried at 50 °C for 2 days, and ground to a particle size of less than 2 mm. It was then macerated in ethyl acetate (EtOAc, 10 L) for 3 days at room temperature. The solvent was subsequently filtered and evaporated under reduced pressure (45 °C, 200 mbar). After three extractions, 127 g of a gummy residue was obtained.

The crude extract was subjected to flash chromatography on silica gel, eluted with a gradient from hexane to EtOAc, yielding 12 fractions (F1–F12). Preliminary phytochemical analysis indicated that fractions F1–F4 predominantly contained fatty acids and pigments, while later fractions exhibited high levels of tannins and sugars. Fraction 9 was selected for further purification as it presented a major compound of interest with minimal interference from secondary metabolites. This fraction was purified by Sephadex LH-20 and column chromatography (40 mesh), eluted with hexane/EtOAc (3:2, v/v), affording 168 mg of cubebin as a white solid. Recrystallization from EtOAc at 4 °C yielded colorless crystals suitable for X-ray diffraction (XRD) analysis.

Given the low yield of cubebin (0.00235% from fresh material), only the purified compound was prioritized for biological evaluation due to logistical and material constraints inherent to an international collaborative study.

### Chemical Characterization of Cubebin

Cubebin was evaluated by one-two-dimensional (1D) and two-dimensional (2D) nuclear magnetic resonance (NMR) spectroscopy. The ^1^H and ^13^C NMR spectra were recorded in CDCl_3_ solution in 5 mm tubes at RT on a Bruker Avance Neo 500 MHz spectrometer (Bruker Biospin GmbH, Rheinstetten, Germany) at 500 (^1^H) and 125 (^13^C) MHz, with the solvent deuterium signal as the shut-off and residual CHCl_3_ (δ = 7.26 ppm for ^1^H) or CDCl_3_ (δ = 77.16 ppm for ^13^C) for internal calibration. All spectra (^1^H, ^13^C, gs-H,H–COSY, edited HSQC, gs-HMBC, and NOESY) were acquired and processed with standard Bruker software TopSpin 4.5.0. High-resolution mass spectra (HRMS) were obtained by EI-TOF (70 eV) using a Waters Micromass instrument.

### Animals, Parasites, and Cell Lines

The life cycle of *A. cantonensis* (NPDN-AC strain) was maintained at the Research Center on Neglected Diseases, Guarulhos University, using either *Biomphalaria glabrata* (freshwater snails) or *Achatina fulica* (terrestrial mollusks) as intermediate hosts, and Wistar rats (*Rattus norvegicus*) as definitive hosts. All animals were maintained under controlled conditions (22 °C, ∼50% humidity) with ad libitum access to food and water.

HaCaT cells (human keratinocytes) were obtained from the Banco de Células do Rio de Janeiro (Duque de Caxias, RJ, Brazil), and Vero cells (monkey kidney epithelial cells) were purchased from the American Type Culture Collection (ATCC CCL-81; Manassas, V). Cells were cultured in DMEM supplemented with 2 mM l-glutamine (Vitrocell), 100 U/mL penicillin, 100 μg/mL streptomycin, and 10% heat-inactivated fetal bovine serum, and maintained at 37 °C in a humidified atmosphere containing 5% CO_2_.^38^

The nematode *C. elegans* (Bristol N2 strain) was maintained on nematode growth medium (NGM) plates seeded with *Escherichia coli* OP5047.^39^

### Antiparasitic Assay with *A. cantonensis* L1 Larvae

First-stage larvae (L1) of *A. cantonensis* were isolated from the feces of infected Wistar rats using the Rugai sedimentation technique,^40^ followed by washing in RPMI 1640 medium containing antibiotics. Approximately 50 larvae were transferred to each well of a 96-well plate. Cubebin and albendazole (positive control) were tested starting at 100 μM using a serial dilution format for EC_50_ determination. Plates were incubated at 21 °C. Untreated larvae served as negative controls. Larval movement was classified as immobile, intermittent, slow, or highly active. Efficacy was defined as ≥60% of larvae becoming immobile after 24 h.^6^

### Antiparasitic Assay with *A. cantonensis* L3 Larvae

Third-stage larvae (L3) of *A. cantonensis* were obtained from infected *A. fulica* *via* artificial digestion in HCl-pepsin solution,^41^ followed by sedimentation using the modified Rugai method.^40^ Approximately 50 larvae were added per well in 96-well plates. Cubebin and albendazole were tested starting at 100 μM in a serial dilution format for EC_50_ estimation. Plates were incubated at 21 °C, and larval viability was assessed at 0 and 24 h using an inverted microscope. Movement was categorized as immobile, intermittent, slow, or highly active. Efficacy was defined as ≥60% immobility after 24 h.

### Lethal Toxicity Assay in *C. elegans*

Synchronized fourth-stage larvae (L4) of *C. elegans* were transferred to 96-well plates containing M9 medium, with approximately 60 larvae per well.^9^ Cubebin was tested at concentrations starting from 1000 μM. Ivermectin (10 μM) served as the positive control, while 0.5% DMSO was used as the negative control. After 24 h of incubation at 21 °C, larval viability was evaluated based on movement.^42^ Toxicity was defined as ≥60% immobility.^43^

### Cytotoxicity Assay

Cytotoxicity was evaluated using the MTT assay as previously described.^44^ Briefly, cells were seeded into 96-well plates (Corning) at a density of 2 × 10^3^ cells per well and exposed to cubebin. The initial concentration tested was 500 μM, followed by 3-fold serial dilutions. Doxorubicin (15 μM) served as the positive control, while 0.5% DMSO was used as negative control. After 24 h of incubation at 37 °C with 5% CO_2_, MTT solution (Sigma) was added, and plates were incubated for an additional 4 h. Absorbance was measured at 595 nm using a spectrophotometer (Epoch, BioTek Instruments, Winooski, VT). Cell viability was expressed as a percentage relative to control wells.^45^ All assays were performed in triplicate and repeated independently three times.

### *In Silico* Analysis

Pharmacokinetic and drug-likeness properties were evaluated using the SwissADME platform.^46^ Chemical structures were drawn using ChemAxon’s Marvin JS sketcher and converted to SMILES format for analysis. Key ADME parameters—absorption, distribution, metabolism, and excretion—were predicted. Drug-likeness was assessed using established filters, including Veber (GlaxoSmithKline), Lipinski (Pfizer), Ghose (Amgen), and Muegge (Bayer), Egan (Pharmacia) criteria. Additionally, the compounds were screened for pan-assay interference structures (PAINS) to evaluate their suitability for drug development.

### Statistical Analysis

EC_50_ and CC_50_ values were calculated from nonlinear sigmoidal dose–response curves using GraphPad Prism version 8.0 (San Diego, CA).^47^ Group differences were analyzed using one-way ANOVA followed by Tukey’s post hoc test. A *P*-value of <0.05 was considered statistically significant.

### Ethic Statement

All procedures involving animals were approved by the Animal Ethics Committee of Guarulhos University (Campus Centro, Guarulhos, SP, Brazil), under protocol number 064/24.

## Supplementary Material



## Notes

Crystal data and crystallographic details for cubebin; illustrations of intermolecular interactions in the solid state; NMR signal assignments with comparisons to literature data; copies of 1D and 2D NMR spectra of cubebin; and bioavailability radar analysis of cubebin are provided (PDF)
