# Supplementary material for: Cubebin, a Lignan Isolated from Drimys andina, Exhibits Potent and Selective Antiparasitic Activity against Angiostrongylus cantonensis
Source: ACS Omega. 2025 Jul 11;10(28):31161–9. doi: 10.1021/acsomega.5c05451 (PMC12290638; doi:10.1021/acsomega.5c05451)
Supplement: Supplementary file 1 [file ao5c05451_si_001.pdf]

Supporting Information

Cubebin, a Lignan Isolated from *Drimys andina,* Exhibits Potent and Selective Antiparasitic Activity Against *Angiostrongylus cantonensis*

Thainá R. Teixeira[1], Bernd Schmidt[2], Eric Sperlich[2], Bruna L. Lemes[1], Monique C. Amaro[1], Rebeca Pérez[3], Camilo Céspedes-Méndez[3], Cecilia Villegas[4], Viviana Burgos[5], Josué de Moraes[1,6*], and Cristian Paz[7*]

[1] Research Center on Neglected Diseases, Guarulhos University, Guarulhos, SP, 07023-070, Brazil;

[2] Institut für Chemie, Universität Potsdam, Karl-Liebknecht-Str. 24-25, Potsdam D-14476, Germany.

[3] Carrera de Química y Farmacia, Facultad de ciencias de la Salud, Universidad Autónoma de Chile, Avenida Alemania 01090, Temuco 4780000, Chile.

[4] Departamento de Ciencias Biológicas y Químicas, Facultad de Recursos Naturales, Universidad Católica de Temuco, Rudecindo Ortega, Temuco 4780000, Chile.

[5] Escuela de Tecnología Médica, Facultad de Salud, Universidad Santo Tomás, Temuco 4780000, Chile.

[6] Research Center on Neglected Diseases, Scientific and Technological Institute, Brazil University, São Paulo, SP, 08230-030, Brazil.

[7] Laboratory of Natural Products & Drug Discovery, Center CEBIM, Department of Basic Sciences, Faculty of Medicine, Universidad de La Frontera, Temuco 4780000, Chile.

***Corresponding authors:**

Josué de Moraes

Núcleo de Pesquisa em Doenças Negligenciadas, Universidade Guarulhos, Praça Tereza Cristina 88, São Paulo, 07023-070, Brazil. E-mail address: moraesnpdn@gmail.com. Tel.: +55 11 24641758.

Cristian Paz

Department of Basic Sciences, Faculty of Medicine, Universidad de La Frontera, Temuco 4780000, Chile. E-mail address: cristian.paz@ufrontera.cl. Tel.: +56452592825.

Table of Contents

1. **NMR spectroscopic analysis of cubebin (Table S1, Figures S1–S7)**
2. **Single-crystal X-ray structure analysis of cubebin (Table S1, Figures S8–S13)**
3. **Bioavailability radar analysis of cubebin (Figure S14)**

1. NMR spectroscopic analysis of cubebin

Table S1. NMR-data of cubebin and comparison with literature data.

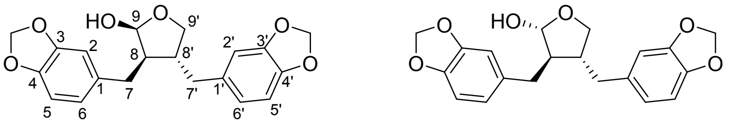

Cubebin (minor anomer) Cubebin (major anomer)

	This work[a]			Literature data for comparison[b]		
position	δ(^{1}H)/ppm	m (J (Hz))	δ(^{13}C)	δ(^{1}H)/ppm	m/J (Hz)	δ(^{13}C)
1-major	--	--	133.5	--	--	133.3
1-minor			134.0			133.8
2-major	6.57	d (1.3)	109.1	6.45	d (1.5)	108.9
2-minor	6.74	d (1.4)	109.1	6.56	d (1.5)	108.9
3-major	--	--	147.8	--	--	147.6
3-minor			147.9			147.7
4-major	--	--	145.9	--	--	145.7
4-minor			145.9			145.7
5-major	6.68	d (8.5)	108.3	6.61	d (8.5)	108.0
5-minor	6.70	d (8.0)	108.4	6.66	d (8.0)	108.2
6-major	6.51	m	121.6	6.44	dd (8.5, 1.5)	121.4
6-minor	6.51	m	121.5	6.52	dd (8.0, 1.5)	121.3
7-major	2.44	m	38.6	2.37	m	38.4
	2.65	dd (14.0, 7.5)		2.60	dd (13.5, 7.5)	
7-minor	2.76	dd (14.0, 10.3)	33.8	2.52	m	33.6
	2.65	m		2.37	m	
8-major	2.13	m	53.3	2.07	m	53.0
8-minor	2.00	m	52.2	1.93	m	51.9
9-major	5.22	m	103.5	5.15	d (4.5)	103.3
9-minor	5.22	m	99.0	5.15	d (1.5)	98.8
1'-major	--	--	134.3	--	--	134.1
1'-minor			134.6			134.5
2'-major	6.52	m	109.3	6.51	d (2.0)	109.1
2'-minor	6.57	m	109.5	6.67	d (2.0)	109.3
3'-major	--	--	147.7	--	--	147.5
3'-minor			147.7			147.5
4'-major	--	--	146.1	--	--	145.9
4'-minor			146.1			145.9
5'-major	6.68	d (8.5)	108.2	6.63	d (7.5)	108.0
5'-minor	6.70	d (8.5)	108.3	6.66	d (8.0)	108.1
6'-major	6.55	dd (7.8, 1.5)	121.9	6.49	dd (7.5, 1.5)	121.7
6'-minor	6.6	d (8.5)	121.8	6.62	dd (8.0, 2.0)	121.6
7'-major	2.60	m	39.4	2.52	m	39.2
	2.59	m		2.50	m	38.8
7'-minor	2.74	m	39.1	2.70	dd (14.0, 10.0)	
	2.60	m		2.50	m	
8'-major	2.15	m	46.0	2.07	m	45.8
8'-minor	2.43	m	43.0	2.37	m	42.9
9'-major	4.00	dd (8.5, 7.3)	72.4	3.93	dd (8.5, 7.0)	72.1
	3.79	t (8.5)		3.73	dd (8.5, 8.0)	

9'-minor	4.10<br>3.58	t (8.3)<br>dd (8.5, 7.5)	72.8	4.03<br>3.50	t (8.5)<br>dd (8.5, 7.5)	72.5
–OCH_2O–	5.94-5.91	in total 4H, m	101.0 (2x)<br>101.0,<br>100.9			100.8 (2x)<br>100.8 (2x)
–OH	not resolved		--	1.72<br>2.86	s, br.<br>s. br.	--

[a] ^{1}H-NMR (500 MHz, $CDCl_3$); ^{13}C-NMR (125 MHz, $CDCl_3$). [b] ^{1}H-NMR (500 MHz, $CDCl_3$); ^{13}C-NMR (125 MHz, $CDCl_3$): de Pascoli, I. C.; Nascimento, I. R.; Lopes, L. M. X. Configurational analysis of cubebins and bicubebin from *Aristolochia lagesiana* and *Aristolochia pubescens*. *Phytochemistry* **2006**, *67*, 735-742.

Figure S1: ^1H NMR (500 MHz, $CDCl_3$) of cubebin

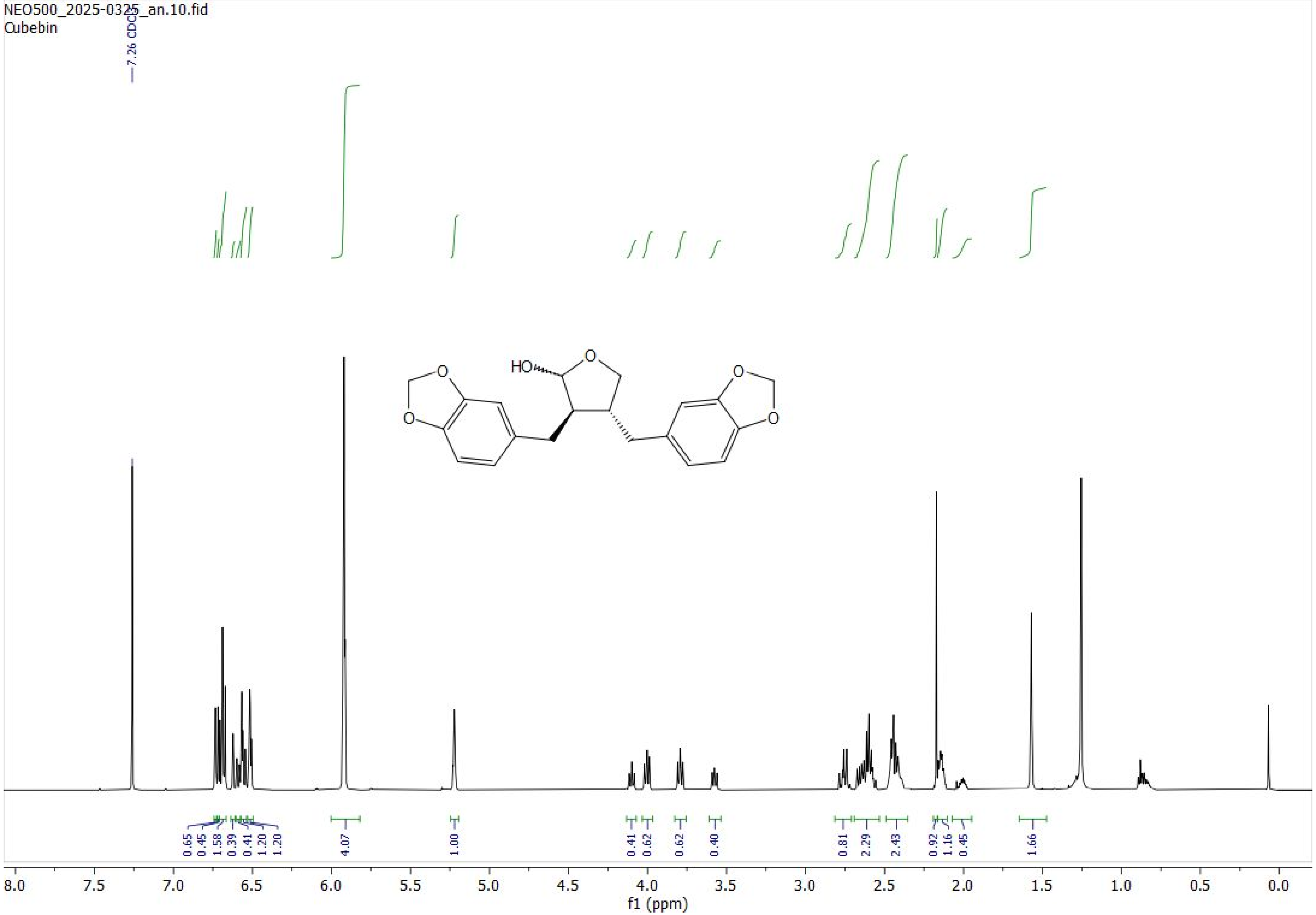

Figure S2: $^{13}C\{^{1}H\}$ NMR (125 MHz, $CDCl_3$) of cubebin

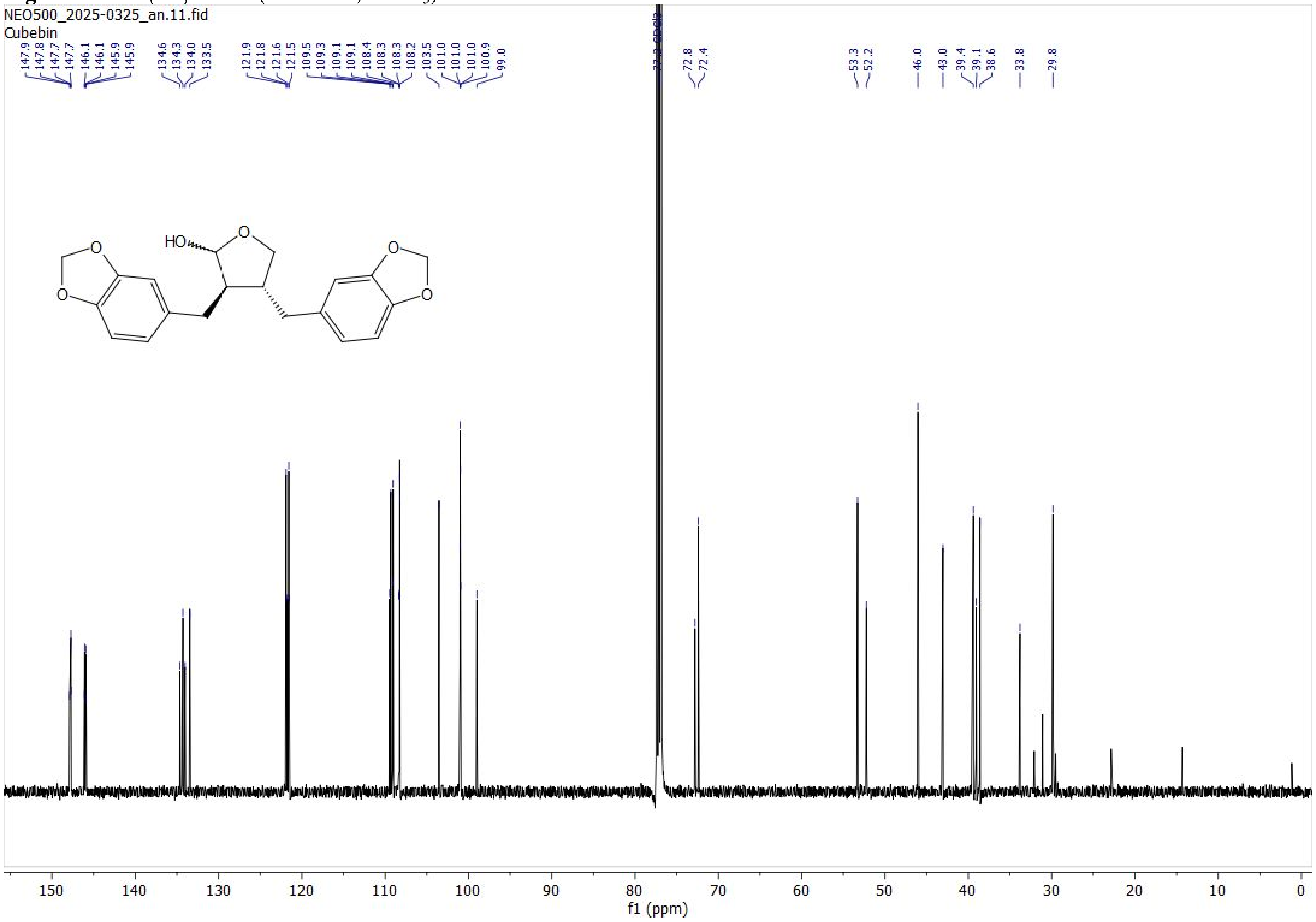

Figure S3: H,H-COSY (500 MHz, $CDCl_3$) of cubebin

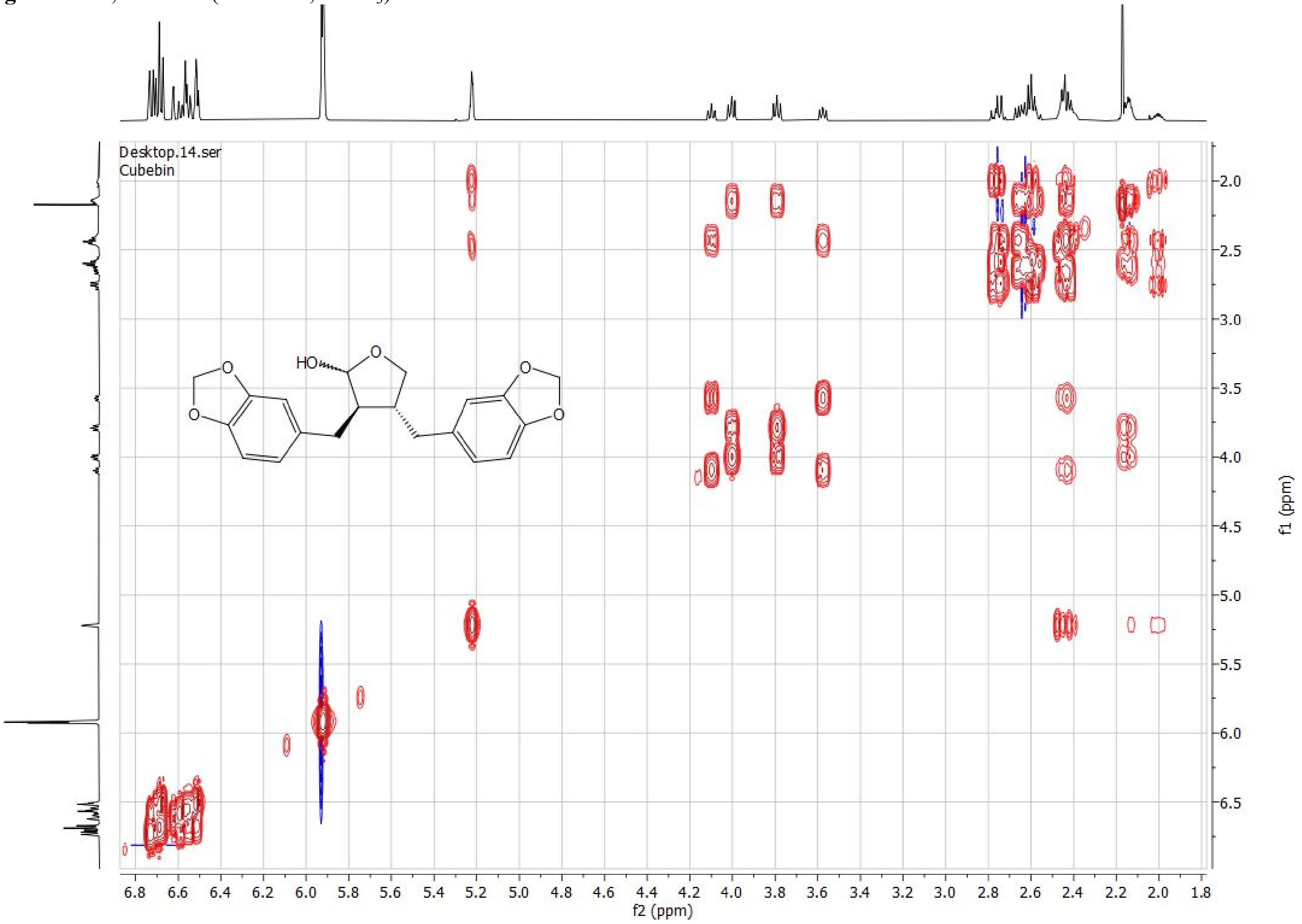

Figure S4: H,H-TOCSY (500 MHz, $CDCl_3$) of cubebin

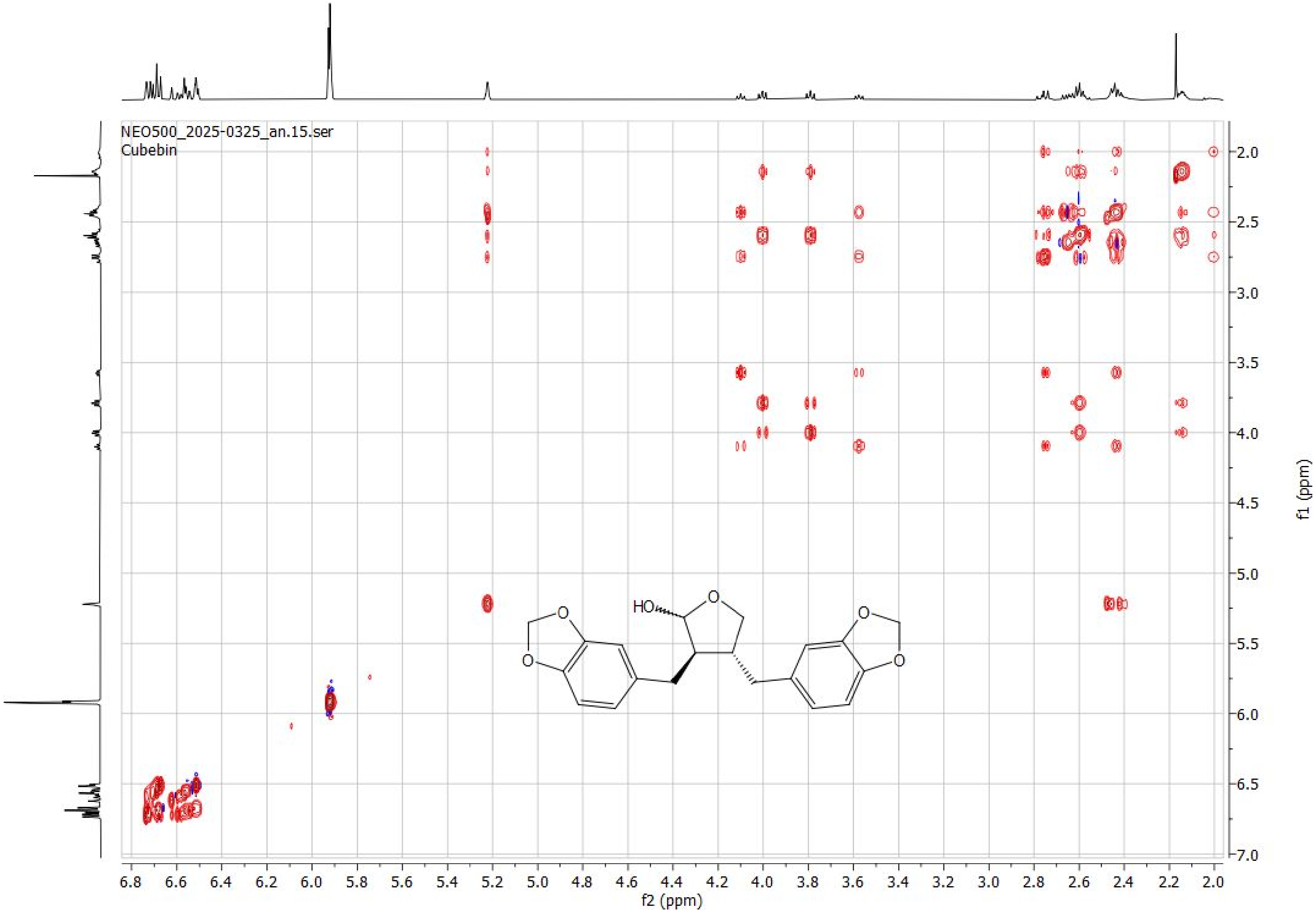

Figure S5: NOESY (500 MHz, $CDCl_3$) of cubebin

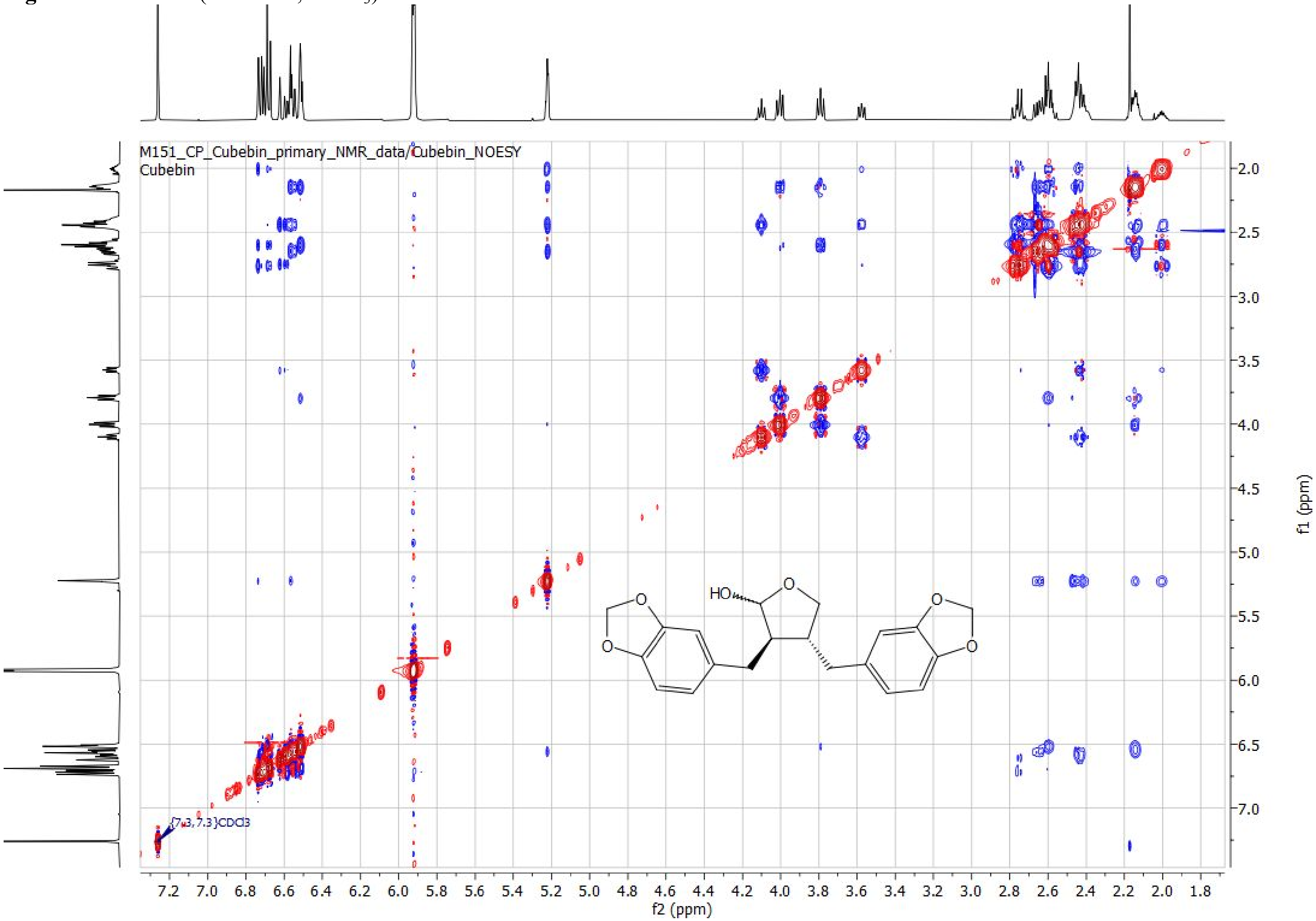

Figure S6: HSQC (500/125 MHz, $CDCl_3$) of cubebin

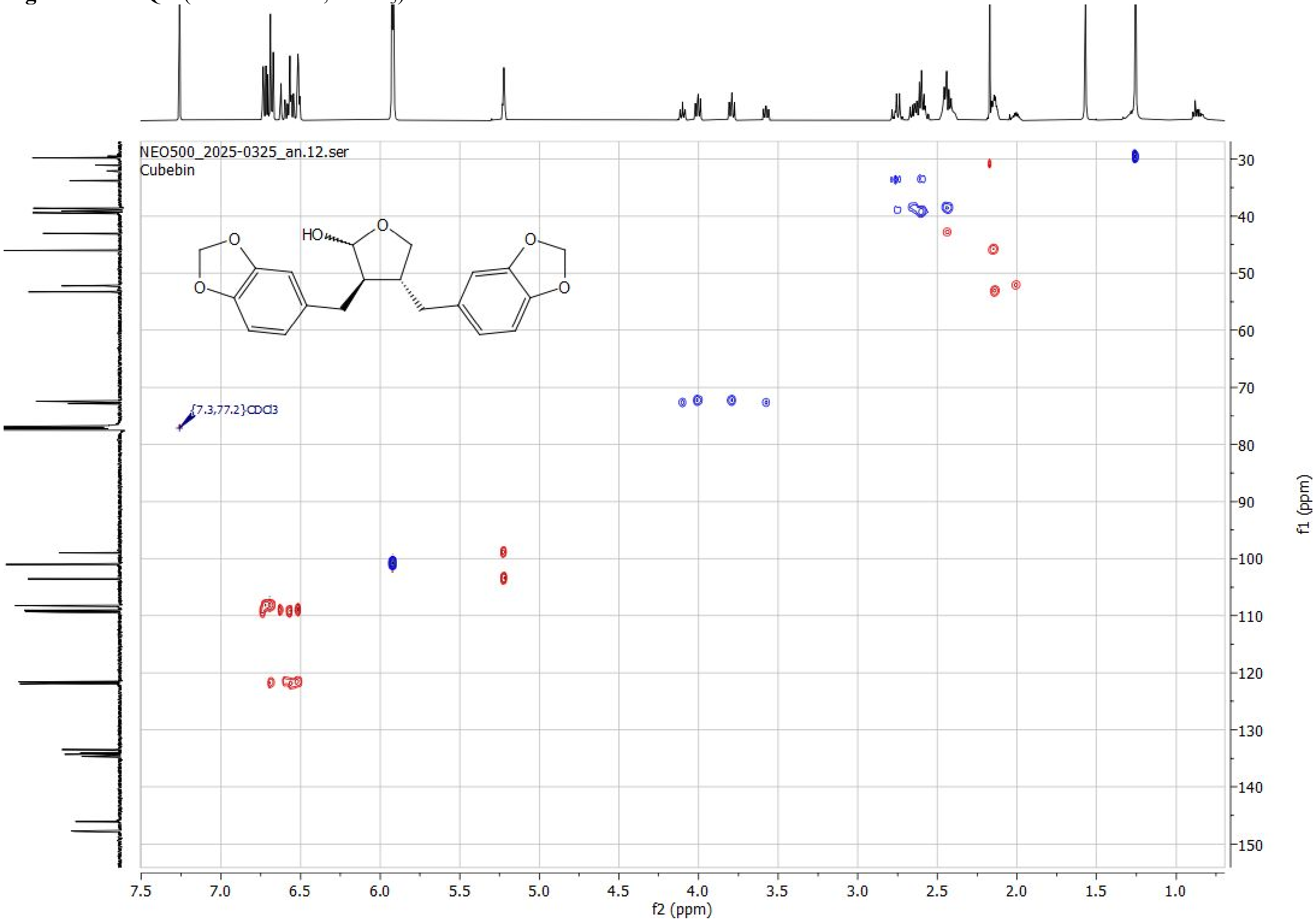

Figure S7: HMBC (500/125 MHz, $CDCl_3$) of cubebin

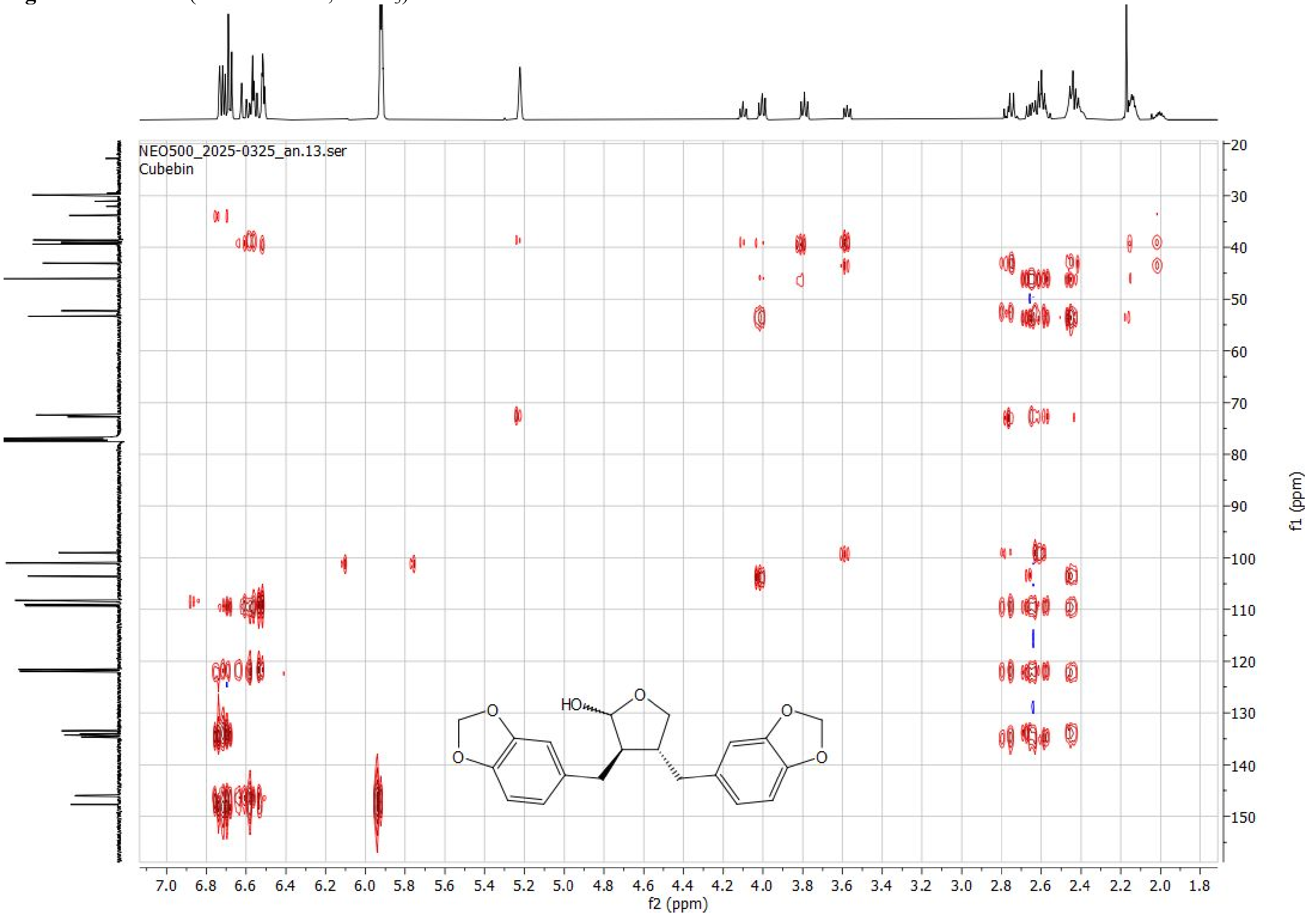

2. Single-crystal X-ray structure analysis of cubebin

General details of X-ray structure analysis

The crystal structure of cubebin has been published as a CSD communication in 2017 (two times) and with an associated publication in 2020 and can be found under the CSD codes GASSIF, GASSIF01, GASSIF02.[1] The intermolecular interactions of the compounds were not discussed in these publications, but will be discussed here.

The crystal structures were determined by single crystal structure analysis. Suitable single crystals were selected using a Leica M205C light microscope and separated with oil. X-ray crystal structure analyses were performed on a Stadivari diffractometer (Stoe) with monochromated Mo-$K\alpha$ radiation (λ = 0.71073 Å). The data correction was performed using the program X-Area.[2] The structures were solved by direct methods and refined against F^2 on all data by full-matrix least-squares using the SHELX suite of programs.[3,4] All non-hydrogen atoms were refined anisotropically; the hydrogen atoms were placed on calculated positions. Table S1 was created using FinalCif.[5] The crystal structure was visualized with Mercury.[6] The data (CCCD 2388202) can be obtained free of charge from The Cambridge Crystallographic Data Centre, http://www.ccdc.cam.ac.uk.

Crystallographic data

Table S8. Crystal data and details of structure refinement for cubebin.

**Compound**	**Cubebin**
CCDC number	2388202
Empirical formula	$C_{19}H_{17}NO_5$
Formula weight	339.33
Temperature [K]	297(2)
Crystal system	monoclinic
Space group (number)	$P2_1/n$ (14)
a [Å]	4.7806(3)
b [Å]	34.5900(18)
c [Å]	9.9830(6)
α [°]	90
β [°]	91.177(5)
γ [°]	90
Volume [Å^3]	1650.45(17)
Z	4
ρ_{calc} [gcm^{-3}]	1.366
μ [mm^{-1}]	0.100
F(000)	712
Crystal size [mm^3]	0.600×0.467×0.200
Crystal colour	colorless
Crystal shape	plate
Radiation	Mo K_α (λ=0.71073 Å)
2θ range [°]	4.71 to 50.00 (0.84 Å)
Index ranges	$-4 \le h \le 5$<br>$-40 \le k \le 40$<br>$-11 \le l \le 11$
Reflections collected	9370
Independent reflections	2827<br>R_{int} = 0.0281<br>R_{sigma} = 0.0206
Completeness to θ = 25°	97.0 %
Data / Restraints / Parameters	2827/0/249
Goodness-of-fit on F^2	1.117
Final R indexes [$I \geq 2\sigma(I)$]	R_1 = 0.0489<br>wR_2 = 0.1122
Final R indexes [all data]	R_1 = 0.0619<br>wR_2 = 0.1180
Largest peak/hole [eÅ^{-3}]	0.14/-0.17

Visualization of the crystal structure and molecular structure for cubebin

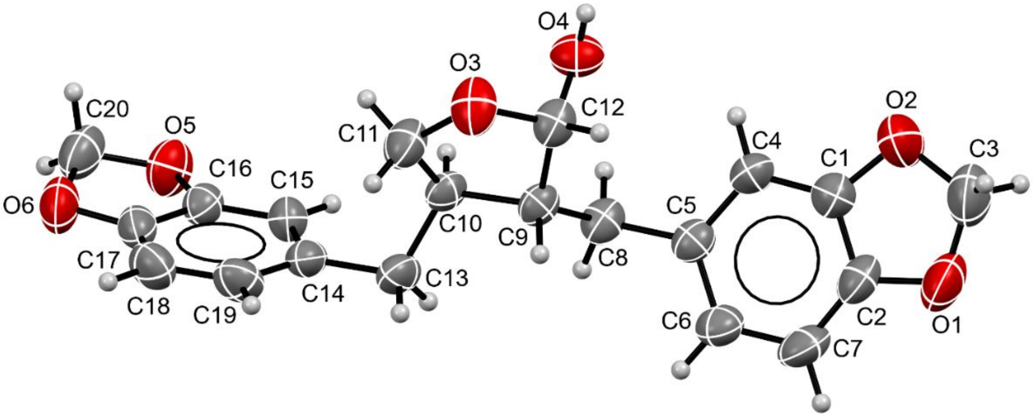

Figure S9: Molecular structure with atom labeling of Cubebin. Displacement ellipsoids are shown at the 50% probability level.

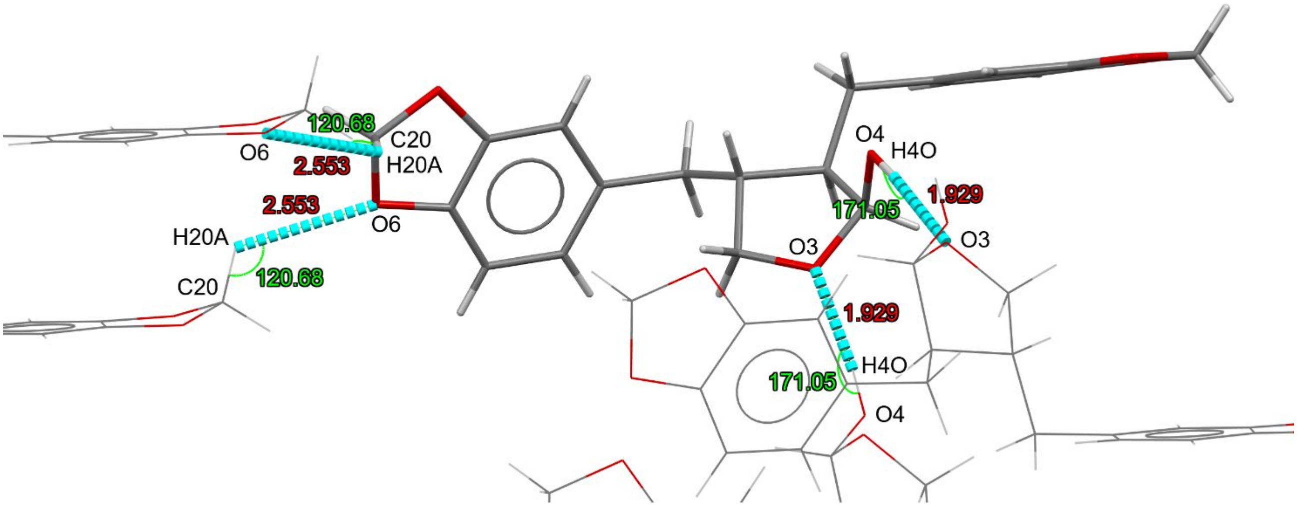

Figure S10: All hydrogen bonds in cubebin with A-H distances up to 2.6 Å (blue dashed lines) and D-H-A angles (green label) of at least 150°.

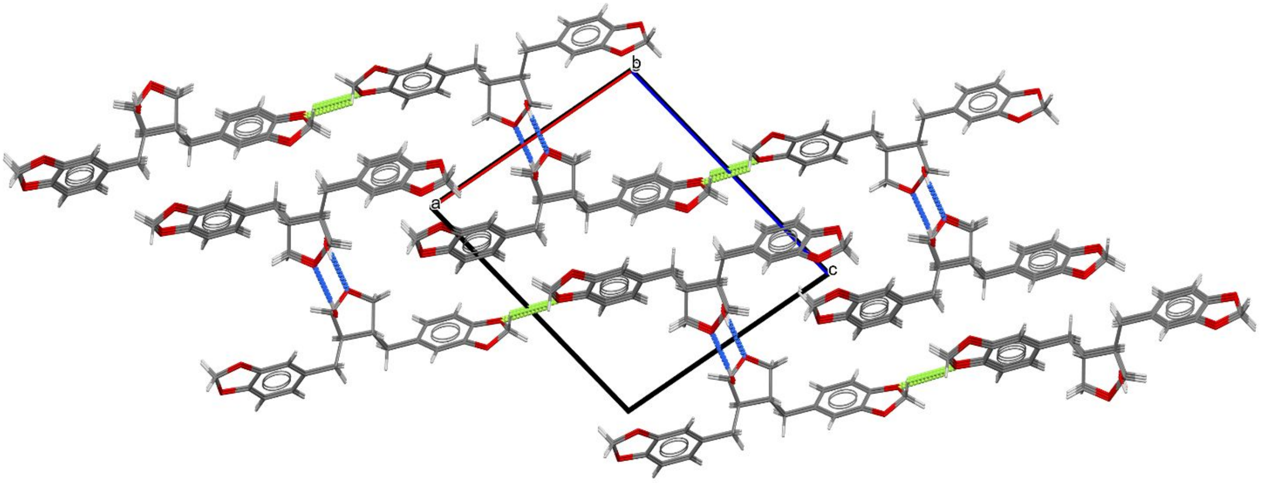

Figure S11: Two-dimensional hydrogen bonding network in cubebin features strong O-H···O bonds (blue dashed lines) along the c axis and weaker C-H···O bonds (green dashed lines) along the a axis, viewed along the b axis.

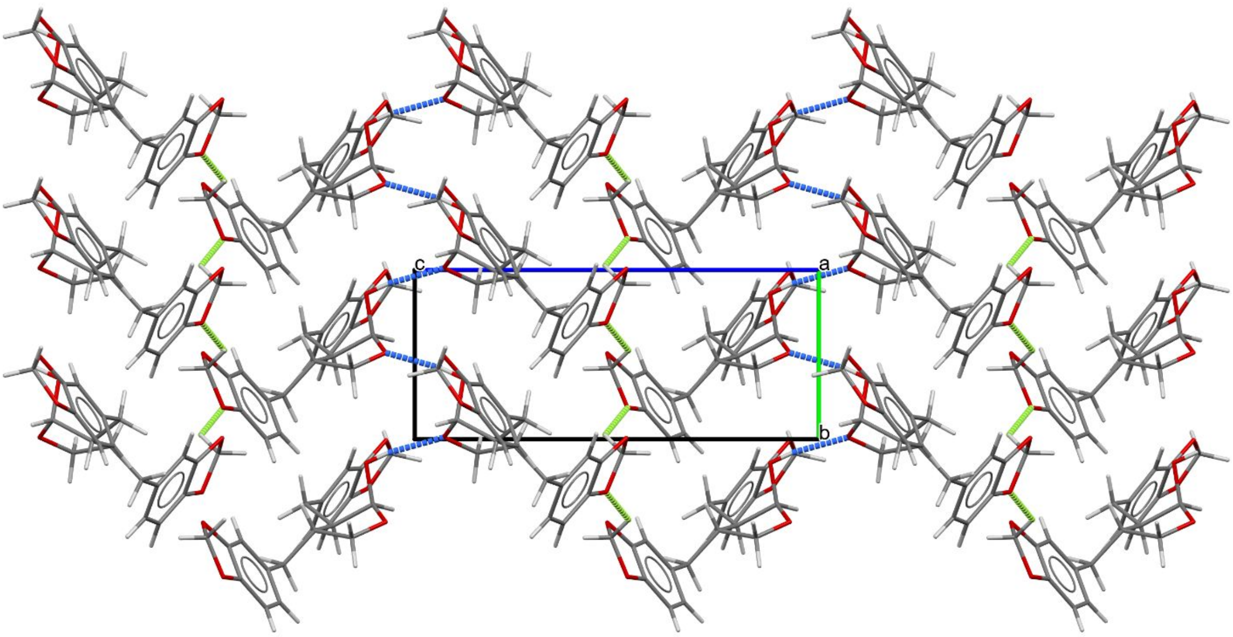

Figure S12: Two-dimensional hydrogen bonding network in **cubebin** features strong O-H···O bonds (blue dashed lines) along the c axis and weaker C-H···O bonds (green dashed lines) along the a axis, viewed along the a axis.

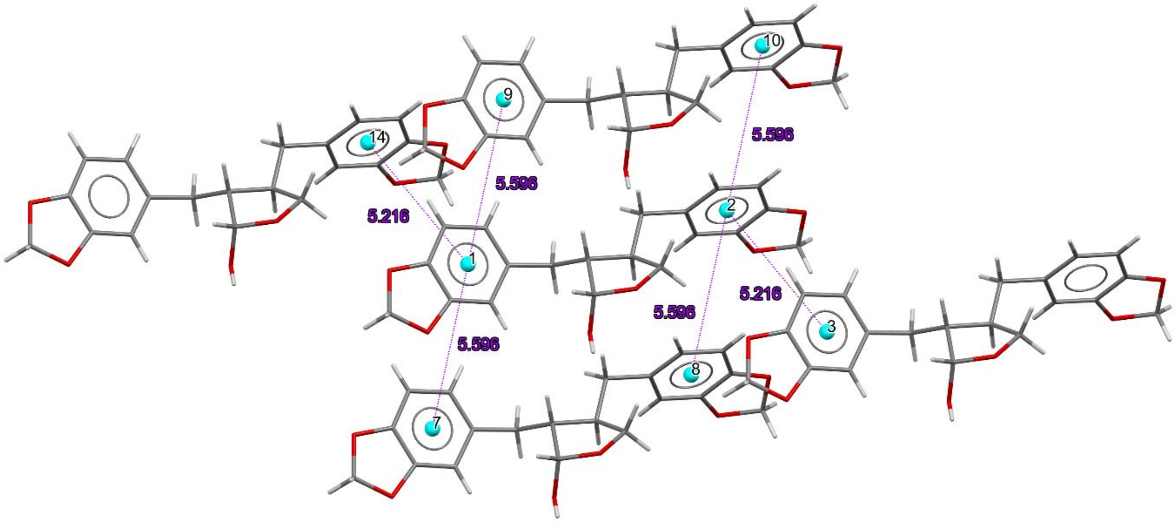

Figure S13: The six strongest stacking interactions (purple dotted lines) between the molecules in cubebin (generated with CSD-Materials - Aromatic Analyser).

Centroid1	Centroid2	Distance	Relative Orientation	Inter-molecular	Score	Assessment
2	8	5,6	0	Yes	6,7	Moderate
2	10	5,6	0	Yes	6,7	Moderate
1	7	5,6	0	Yes	6,5	Moderate
1	9	5,6	0	Yes	6,5	Moderate
1	14	5,22	64,64	Yes	6,5	Moderate
2	3	5,22	64,64	Yes	6,5	Moderate
2	22	5,41	63,34	Yes	6,1	Moderate
2	26	5,41	63,34	Yes	6,1	Moderate
1	12	5,54	64,64	Yes	5,9	Moderate
2	5	5,54	64,64	Yes	5,9	Moderate
1	27	6,15	65,64	Yes	3,6	Moderate
1	29	6,15	65,64	Yes	3,6	Moderate

Figure S6: The strongest stacking interactions between the molecules in cubebin (generated with CSD-Materials - Aromatic Analyser).

3. Bioavailability radar analysis of cubebin

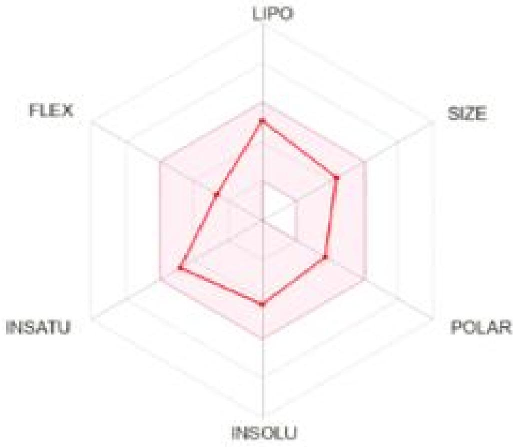

Figure S14. Bioavailability radar plot of cubebin. The radar plot illustrates cubebin's predicted oral bioavailability based on six key parameters: lipophilicity (LIPO), size, polarity (POLAR), solubility (INSOLU), flexibility (FLEX), and saturation (INSATU). All values fall within the optimal range, supporting cubebin's potential as an orally bioavailable drug candidate.

References

(1) Macedo, A.; Martorano, L.; Ferreira de Albuquerque, A. C.; Fiorot, R.; Carneiro, J.; Campos, V.; Vasconcelos, T.; Valverde, A.; Moreira, D.; Martins dos Santos Junior, F. Absolute Configuration of (−)-Cubebin, a Classical Lignan with Pharmacological Potential, Defined by Means of Chiroptical Spectroscopy. *J. Braz. Chem. Soc.* **2020**, *31*, 2030-2037.

(2) STOE & Cie GmbH (2018) X-Area. software package for collecting single-crystal data on STOE area-detector diffractometers, for image processing, for the correction and scaling of reflection intensities and for outlier rejection. STOE & Cie GmbH, Darmstadt.

(3) Sheldrick, G. Crystal structure refinement with SHELXL. *Acta Cryst. C* **2015**, *C71*, 3-8.

(4) Sheldrick, G. A short history of SHELX. *Acta Cryst. A* **2008**, *A64*, 112-122.

(5) FinalCif. https://dkratzert.de/finalcif.html.

(6) Macrae, C. F.; Sovago, I.; Cottrell, S. J.; Galek, P. T. A.; McCabe, P.; Pidcock, E.; Platings, M.; Shields, G. P.; Stevens, J. S.; Towler, M.; Wood, P. A. Mercury 4.0: from visualization to analysis, design and prediction. *J. Appl. Cryst.* **2020**, *53*, 226-235.
